# Experimental *Chlamydia gallinacea* infection in chickens does not protect against a subsequent experimental *Chlamydia psittaci* infection

**DOI:** 10.1186/s13567-021-01011-y

**Published:** 2021-11-20

**Authors:** Marloes Heijne, Jeanet van der Goot, Herma Buys, Annemieke Dinkla, Hendrik Jan Roest, Lucien van Keulen, Ad Koets

**Affiliations:** 1 Department of Bacteriology, Host-Pathogen Interaction and Diagnostics Development, Wageningen Bioveterinary Research, Lelystad, The Netherlands; 2 Department of Diagnostics and Crisis Organisation, Wageningen Bioveterinary Research, Lelystad, The Netherlands; 3 Current Affiliation: Directorate Animal Supply Chain and Animal Welfare, Ministry of Agriculture, Nature and Food Quality, The Hague, The Netherlands; 4 Department of Population Health Sciences, Faculty of Veterinary Medicine, Utrecht University, Utrecht, The Netherlands

**Keywords:** *Chlamydia*, Cross immunity, Cross protection, Chickens

## Abstract

*Chlamydia psittaci* was considered the predominant chlamydial species in poultry until *Chlamydia gallinacea* was discovered in 2009. *C. psittaci* is a zoonotic obligate intracellular bacterium reported in more than 465 bird species including poultry. In poultry, infections can result in asymptomatic disease, but also in more severe systemic illness. The zoonotic potential of *C. gallinacea* has yet to be proven. Infections in poultry appear to be asymptomatic and in recent prevalence studies *C. gallinacea* was the main chlamydial species found in chickens. The high prevalence of *C. gallinacea* resulted in the question if an infection with *C. gallinacea* might protect against an infection with *C. psittaci*. To investigate possible cross protection, chickens were inoculated with *C. gallinacea* NL_G47 and subsequently inoculated with either a different strain of *C. gallinacea* (NL_F725) or *C. psittaci*. Chickens that had not been pre-inoculated with *C. gallinacea* NL_G47 were used as a *C. gallinacea* or *C. psittaci* infection control. In the groups that were inoculated with *C. psittaci*, no difference in pharyngeal or cloacal shedding, or in tissue dissemination was observed between the control group and the pre-inoculated group. In the groups inoculated with *C. gallinacea* NL_F725, shedding in cloacal swabs and tissues dissemination was lower in the group pre-inoculated with *C. gallinacea* NL_G47. These results indicate previous exposure to *C. gallinacea* does not protect against an infection with *C. psittaci*, but might protect against a new infection of *C. gallinacea*.

**Supplementary Information:**

The online version contains supplementary material available at 10.1186/s13567-021-01011-y.

## Introduction

*Chlamydia gallinacea* and *Chlamydia psittaci* belong to the *Chlamydiaceae*, a family of obligate intracellular bacteria that currently consists of one genus and 14 species [1]. Until 2009, *C. psittaci* was considered the predominant chlamydial species in poultry. *C. psittaci* is ubiquitous and has been reported in several mammalian species and more than 465 bird species including poultry [2]. Infections can remain asymptomatic, but also result in severe systemic illness and mortality depending on the chlamydial strain, host, host age and (environmental) stressors [3]. Moreover, *C. psittaci* has a known zoonotic potential; infections in humans can ultimately result in severe pneumonia [3].

Since 2009, atypical *Chlamydia* species were identified in poultry and in 2014 classified as *C. gallinacea* after additional sequencing [4, 5]. *C. gallinacea* is most closely related to *C. avium*, which was formerly identified as an atypical *Chlamydia* species in parrots and pigeons. *C. gallinacea* is highly prevalent in chickens [6–8]. Experimental infections with *C. gallinacea* do not result in clinical signs of disease [9, 10], but can lead to production loss such as reduced weight gain [8]. There is currently no microbiological evidence of a zoonotic potential of *C. gallinacea*, although *C. gallinacea* has been considered the causative agent in cases of pneumonia in slaughterhouse workers [5, 6].

In a Dutch cross sectional study in 2018, *C. gallinacea* was detected by qPCR in pooled faecal samples at 71 of the 151 investigated layer farms. *C. psittaci* was not detected in any sample from these farms [6]. This was unexpected, since a Belgian study, a bordering country of the Netherlands, reported 6/7 broiler breeder, 7/7 broiler and 5/5 layer farms qPCR and culture positive for *C. psittaci* in pharyngeal swabs in 2014 [11]. Other studies in Belgium and Northern-France from 2010 and 2013 also reported a high prevalence of *C. psittaci* determined with qPCR, culture (on pharyngeal swabs and tissues) and/or serology [12, 13]. These differences might be a result of differences in methodology. In the Dutch study [6], pooled faecal samples were collected for PCR detection while in other studies individual pharyngeal swabs and tissue samples were collected for culture and PCR confirmation [11–13]. It is known that pharyngeal swabs are a more sensitive method to detect *C. psittaci* than the collection of cloacal swabs or faecal samples [14].

With the current understanding of *C. gallinacea* in poultry, a high seroprevalence of *C. psittaci*, might also be explained by possible cross reactive antibodies as a major outer membrane protein (MOMP) based *C. psittaci* ELISA was used [13]. Cross reactive antibodies between chlamydial species are known to occur because of the close structural similarity among some of the major surface antigens such as MOMP [15] and could potentially result in cross protection against multiple *Chlamydia* species. This has been observed in mice which were vaccinated with a live strain of *Chlamydia abortu*s and subsequently challenged with *Chlamydia pecorum*. Vaccination with *C. abortus* resulted in reduced placental colonization of *C. pecorum* [16]. Therefore, cross protection may offer an alternative explanation why *C. psittaci* was not detected in the Dutch prevalence study [6] or atypical *Chlamydia* (later classified as *C. gallinacea*) were not detected in Belgian chickens broilers [11]. We hypothesised that the high prevalence of *C. gallinacea* in Dutch layers resulted in herd immunity against *C. psittaci* due to possible cross protection.

To investigate the hypothesis of possible cross protection, chickens were inoculated with *C. gallinacea* NL_G47 and, after five weeks, inoculated with either a different strain of *C. gallinacea* (NL_F725) or with a strain of *C. psittaci*. These treatments were compared to single exposure with either *C. gallinacea* (NL_F725) or *C. psittaci.* Reduced shedding or tissue dissemination in the groups that had been pre-inoculated with *C. gallinacea* NL_G47 would be an indication of possible cross protection between *C. gallinacea* strains and/or *C. psittaci*. Cross protection between *C. gallinacea* and *C. psittaci* could be a beneficial scenario from a one health perspective, because infections with *C. gallinacea* seem relatively harmless for poultry and *C. gallinacea* has no proven zoonotic potential thus far.

## Materials and methods

### Ethical statement

The animal experiment was conducted in accordance with the national regulations on animal experimentation. The project was approved by the Dutch Central Authority for Scientific Procedures on Animals (CCD) (permit number AVD4010020173926).

### Inocula

*Chlamydia gallinacea* NL_G47 and NL_F725 were isolated from caecal material from laying hens as described earlier [17]. *Chlamydia psittaci* strain NL_Borg is an in-house reference strain of *ompA* genotype D and is closely related to the turkey outbreak strain *C. psittaci* NJ1 with only 65 Single Nucleotide Polymorphisms (SNPs) [17]. All strains were passaged three times in the yolk sac of embryonated SPF chicken eggs and stored at -80 °C in a 20% yolk sac suspension in Sucrose Phosphate Glutamate (SPG) until inoculation [18]. The infectious dose of the suspensions was calculated via egg titration experiments and expressed as the Egg Infectious Dose 50 (EID_50_) [17, 19, 20].

### Animals and housing

A total of 48 five-week-old Specified Pathogen Free (SPF) White Leghorn layers were obtained from Royal GD (Deventer, the Netherlands). *Chlamdiaceae* are not included in standard SPF testing, therefore 10 cloacal swabs from layers of the mother flock were collected. All swabs tested qPCR negative for *Chlamydia* spp. before the chickens were delivered. All chickens had a seven-day acclimatization period prior to the first inoculation.

After arrival the hens were housed in groups on sawdust bedding in temperature-controlled rooms under optimal light conditions and humidity. Feed and water were provided ad libitum. Control chickens or chickens infected with *C. gallinacea* were housed in veterinary biosafety level 2 (vBSL 2) facilities and chickens infected with *C. psittaci* were housed in biosafety level 3 (BSL 3) facilities at Wageningen Bioveterinary Research (WBVR, Lelystad, the Netherlands).

### Experimental design

**Figure 1:**
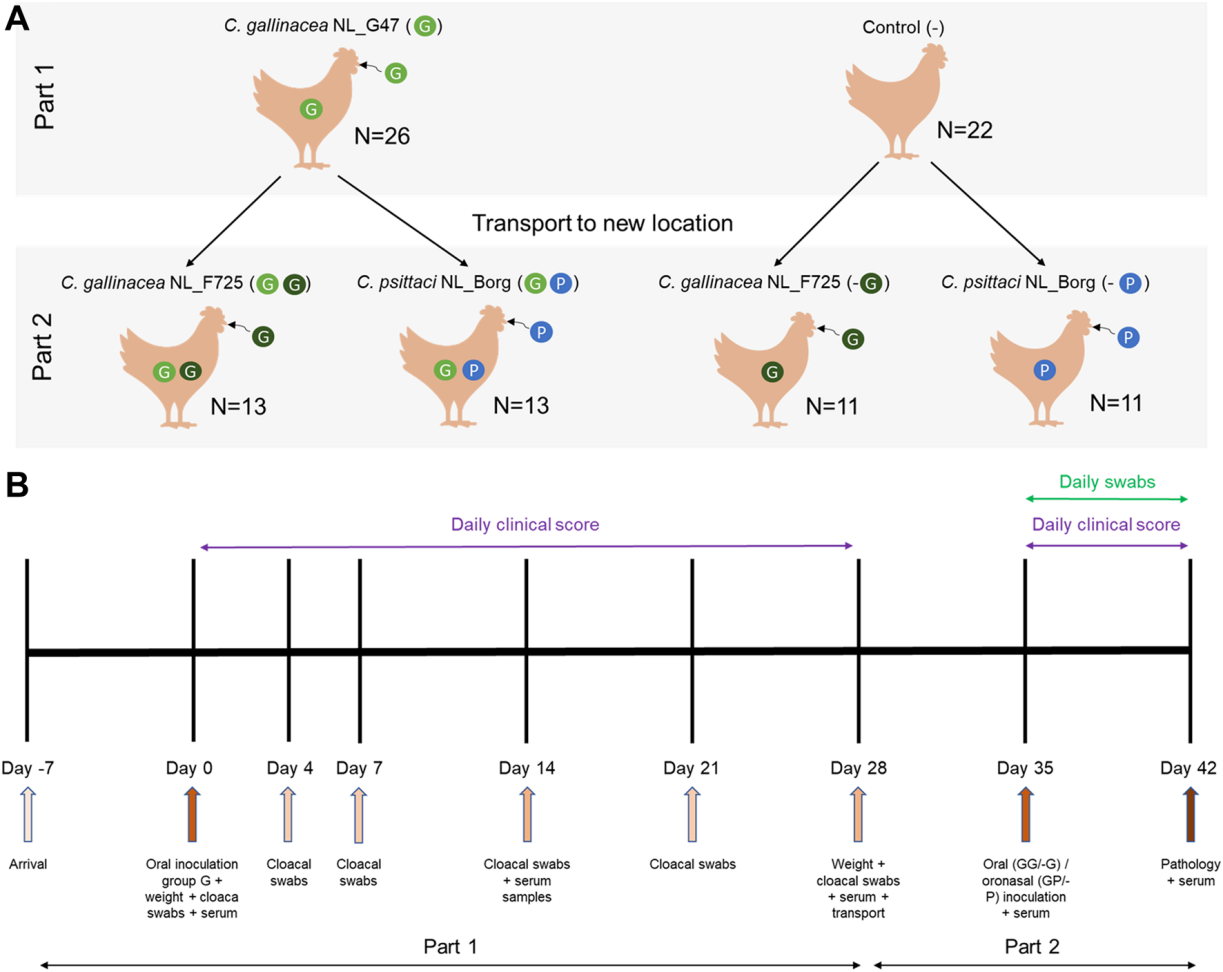
**Experimental design. A** shows the experimental setup with group size and type of inoculum (coloured bacteria) and **B** the timeline of the experiment with sampling moments. From day 35 to day 42 daily pharyngeal and cloacal swabs were collected in the *C. psittaci* inoculated group GP and -P. In group GG and -G only daily cloacal swabs were collected.

The experiment consisted of two parts as shown in Figure 1A. In the first part 26 randomly selected chickens were orally inoculated with *C. gallinacea* NL_G47 seven days after arrival. The remaining twenty-two chickens were not inoculated and served as a control group. Both groups were housed seperately and chickens were numbered randomly. At day 28, after the first inoculation, the groups were transported to a new location with BSL 3 facilities. Both groups were transported separately to prevent cross contamination.

At the new location the control group and the infected group were further subdivided in two groups (resulting in four experimental groups, Figure 1A). The chickens were allocated to the groups alternately by number. At day 35, chickens were either inoculated with *C. gallinacea* strain NL_F725 or with the *C. psittaci* NL_Borg strain. For *C. gallinacea* inoculation was performed orally, because the fecal–oral route is the main route of transmission [9]. For *C. psittaci* inoculation was performed oro-nasally, because both the respiratory and oral route have been described [21]. At day 42, the animals were sacrificed (see Figure 1B).

The inoculations were performed with a 1 mL syringe (Terumo Europe N.V., Leuven, Belgium) and an oral gavage needle (18Gx1,5", Terumo Europe N.V.). For the oro-nasal inoculation, chickens first received one droplet of the suspension in one nostril after which the remaining suspension was inoculated orally. At the first inoculation with *C. gallinacea* NL_G47 chickens received 0.5 mL of a yolk suspension of NL_G47 with an infectious dose of 10^5.9^ EID_50_ per mL. At the second round chickens were inoculated with either 0.5 mL of a yolk suspension of NL_F725 or with 0.5 mL of a yolk suspension of *C. psittaci* NL_Borg, both with an infectious dose of 10^5.4^ EID_50_ per mL. The inoculation dose was confirmed by back-titration and the infectious dose was within a range of one log step of the initial dose.

In the first part of the experiment, the weight of the animals was recorded at day 0 (before inoculation) and day 28. During the whole experiment (42 days) clinical signs were recorded daily according to a clinical scoring card (Additional file 1). In the first part of the experiment, cloacal swabs (Puritan HydraFlock sterile swab, ITK Diagnostics BV, Uithoorn, the Netherlands) were collected at day 0, 4, 7, 14 and 28. In the second part, cloacal swabs were collected daily from day 35 until day 42 in all groups. In the *C. psittaci* exposed groups, additional pharyngeal swabs were collected daily, because for *C. psittaci* pharyngeal swabs might be a more sensitive method to measure shedding [14]. In the *C. gallinacea* groups no pharyngeal swabs were collected based on earlier results that showed shedding in cloacal swabs was higher [10]. In the first part, serum samples were collected from the brachial wing vein at day 0, day 14, day 28 and, in the second part, at day 35. At day 42 serum was collected by cardiac puncture. All samples at day 0 were collected prior to inoculation to confirm the absence of a current *Chlamydia* infection. A timeline of the experiments including sampling moments is given in Figure 1B.

At the end of the experiment the chickens were euthanized by maximum blood collection via heart puncture, under anesthesia by intramuscular injection of a mixture of 30 mg/kg ketamine (Ketamine 10%, Alfasan Diergeneesmiddelen B.V., Woerden, the Netherlands) and 10 mg/kg xylazine (Sedamun, Dechra Veterinary Products, Bladel, the Netherlands).

### Necropsy

At necropsy, carcasses were opened on a clean plastic sheet which was replaced after each necropsy. To prevent cross contamination, new sterile instruments and petridishes were used for every tissue sample. Tissue samples (approximately 0.5 cm^3^) were collected from the airsac, lung, liver, spleen, kidney, jejunum, ileum, caecum, caecal tonsil and colon. Samples for qPCR were collected in 1 mL SPG in Lysing Matrix D tubes (MP Biomedicals, Brussels, Belgium) and ribolysed (2 × 20 s at 4 m/s) before storage at −80 °C. Tissue samples for histology and immunohistochemistry were collected in 10% neutral buffered formalin and routinely processed into paraffin blocks.

### PCR analyses

Swabs were immersed in 1.5 mL PBS 13 (BM014, WBVR) and thoroughly vortexed (10 s) to suspend the sample from the swab. From the swab or tissue suspension, 200 µL was used for DNA extraction. Swabs and tissue suspensions from the BSL 3 lab were heated for 30 min at 99 °C before cell lysis to prevent transfer of infectious material from the containment area. A prior pilot experiment established that the heating step did not influence the qPCR outcome. DNA extraction was performed with a MagNA Pure LC total Nucleic Acid Isolation kit in the MagNA Pure® system (Roche Diagnostics, Almere, the Netherlands) according to instructions provided by the manufacturer. All DNA samples were tested with a *Chlamydiaceae* qPCR targeting the 23S rRNA, as previously described [6, 22]. Samples from chickens that were exposed to *C. psittaci* were also tested with a specific *C. psittaci* qPCR targeting the *ompA* gene according to methods published previously [23].

### Histology and immunohistochemistry

Formalin fixed tissue samples were cut into 4 μm sections and collected on positively charged glass slides (SuperfrostPlus®, Thermo Fisher Scientific, Breda, the Netherlands). Sections were then stained with haematoxylin–eosin (HE) or immunostained with a polyclonal anti-*Chlamydia* antibody (LifeSpan BioSciences, Cat# LS-C85741-1000, RRID:AB_1813851) or a monoclonal anti-*Chlamydia* antibody (MyBioSource, Cat# MBS830551). Epitope retrieval of the formalin fixed sections consisted of proteolysis induced epitope retrieval for the polyclonal antibody (0.1% protK in TBS for 30 min at 37 °C) and heat induced epitope retrieval (citrate buffer, pH 6.0, 121 °C for 5 min) for the monoclonal antibody. Anti-rabbit or anti-mouse HRP conjugated polymer was used as a secondary antibody (Invitrogen, Carlsbad, USA) and DAB + as substrate (Dako, Agilent, Santa Clara, USA). Sections were counterstained with Mayer’s hematoxylin and mounted permanently. Photographs were taken with an Olympus BX51 microscope equipped with a high-resolution digital camera.

### Serology

Serum samples were tested with an in-house ELISA using a commercially available mix of *Chlamydia abortus* and *Chlamydia trachomatis* antigens (Institut Virion\Serion GmbH, Würzburg, Germany), because specific serological tests for *C. gallinacea* are currently not commercially available. An antigen coating solution was prepared with a final concentration of 4 µg / mL of each antigen in bicarbonate coating buffer with pH 9.6 (BM112, WBVR, Lelystad, the Netherlands). Ninety-six-well microtiter plates (Nunc MaxiSorp™, Thermo Fisher Scientific) were coated overnight at 37 °C with 100 µL per well in coating buffer. Following six washes with 0.05% Tween® 80, the plates were blocked with 190 µL per well of 5% skimmed-milk powder (Elk, FrieslandCampina, Amersfoort, the Netherlands) in Tris-buffered saline with 0.1% Tween® 20 detergent (TBST, BM309, WBVR) for 60 min at room temperature (RT). The plates were washed as described above, subsequently 100 µL of chicken serum per well (diluted 1:500 in 5% skimmed milk powder-TBST) was added and the plates were incubated for 60 min at 37 °C. After further washing, 100 µL of goat anti-chicken IgY(H + L)-HRP (Southern Biotech, Birmingham, USA), diluted 1:6000 in 5% skimmed milk powder-TBST) was added per well, and the mixture was incubated for 60 min at 37 °C. Again six washes with 0.05% Tween® 80 were performed and one wash with Super-Q® water. Bound antibody was detected with TMB One component HRP Microwell substrate (TMBW-1000–01, SurModics, Minnesota, USA). The reaction was terminated after 10 min by the addition of 100 µL 0.5 M sulfuric acid. The optical density (OD) was measured at 450 nm on a Thermo Labsystems Multiskan RC microplate reader (Thermo Fisher Scientific).

Per plate, two plate controls were included with two wells per control. In one control, no serum and no conjugate was added to the wells, in the other control no serum was added. All obtained chicken sera were tested in one batch and the individual OD values were corrected for plate differences by subtracting the mean OD value of the plate control (without serum but with conjugate). Furthermore, a *C. gallinacea* positive and negative control were included, originating from previous chicken experiments [10].

### Statistics

Groups were compared using a linear mixed model with Ct value as outcome, for the swabs Day and Group were fixed effects and Chicken a random effect. For the model with the organs, Organ and Group were fixed effects and Chicken a random effect. Models with and without Group were compared by the likelihood ratio test. Analyses were performed in R [24], using the package lme4.

## Results

To investigate possible cross protection chickens were first inoculated with *C. gallinacea* NL_G47 (part 1 of the study) and after five weeks inoculated with *C. gallinacea* NL_F725 or *C. psittaci* NL_Borg (part 2 of the study). During part 1 the control group was not inoculated (see experimental design in Figure 1).

### Part 1: primary inoculation with *C. gallinacea* NL_G47

**Figure 2:**
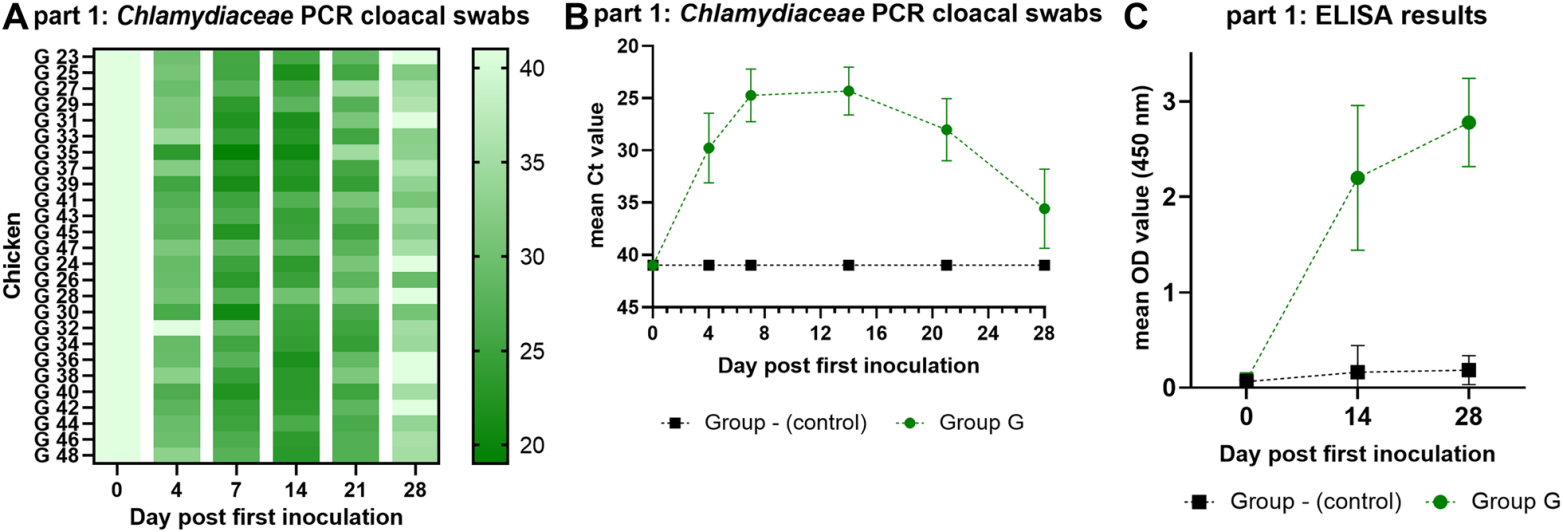
**qPCR results of cloacal swabs (A and B) and ELISA results of serum samples (C).** In **A** results of individual cloacal swabs of group G in the *Chlamydiaceae* qPCR per timepoint are depicted in a heatmap. The darker the colour, the lower the Ct value as shown in the colour scale at the right side. Ct values > 40 or no qPCR signal are shown as Ct 41. The results of the negative control group (-) are not shown. **B** shows the mean Ct value of the cloacal swabs in time per group (of the *Chlamydiaceae* qPCR). The error bar indicates the SD. On the Y-axis the cycle treshold (Ct) value is depicted. The Y-axis has been rotated and Ct values > 40 or no qPCR signal are shown as Ct 41. In **C** the mean OD (450 nm) value of the serum samples per group per timepoint is shown. The error bar indicates the SD.

The group that was inoculated with C. *gallinacea* NL_G47 (group G) in part 1 of the experiment was successfully infected (Figures 2A–C). All cloacal swabs tested positive in the *Chlamydiaceae* qPCR at day 7 and after day 14 shedding declined as shown in Figures 2A, B. Before transport at day 28, 19 / 26 (73%) cloacal swabs were qPCR positive (i.e. Ct < 40) with a mean Ct of 35.6. Furthermore, a rise in antibody titre in the ELISA was observed (Figure 2C). The uninfected control group (-) remained qPCR negative in cloacal swabs and seronegative in the ELISA (Figures 2A–C). During the first 28 days after inoculation no clinical signs, nor a difference in weight was observed in the controls and *C. gallinacea* inoculated chickens (Additional file 2).

### Part 2: secondary inoculations

In part 2 both groups were split resulting in four experimental groups. Two groups (GG and -G) were inoculated with *C. gallinacea* NL_F725 and two groups (GP and -P) were inoculated with *C. psittaci* (Figure 1A).

#### Secondary inoculation with *C. psittaci* (group GP and -P)

**Figure 3:**
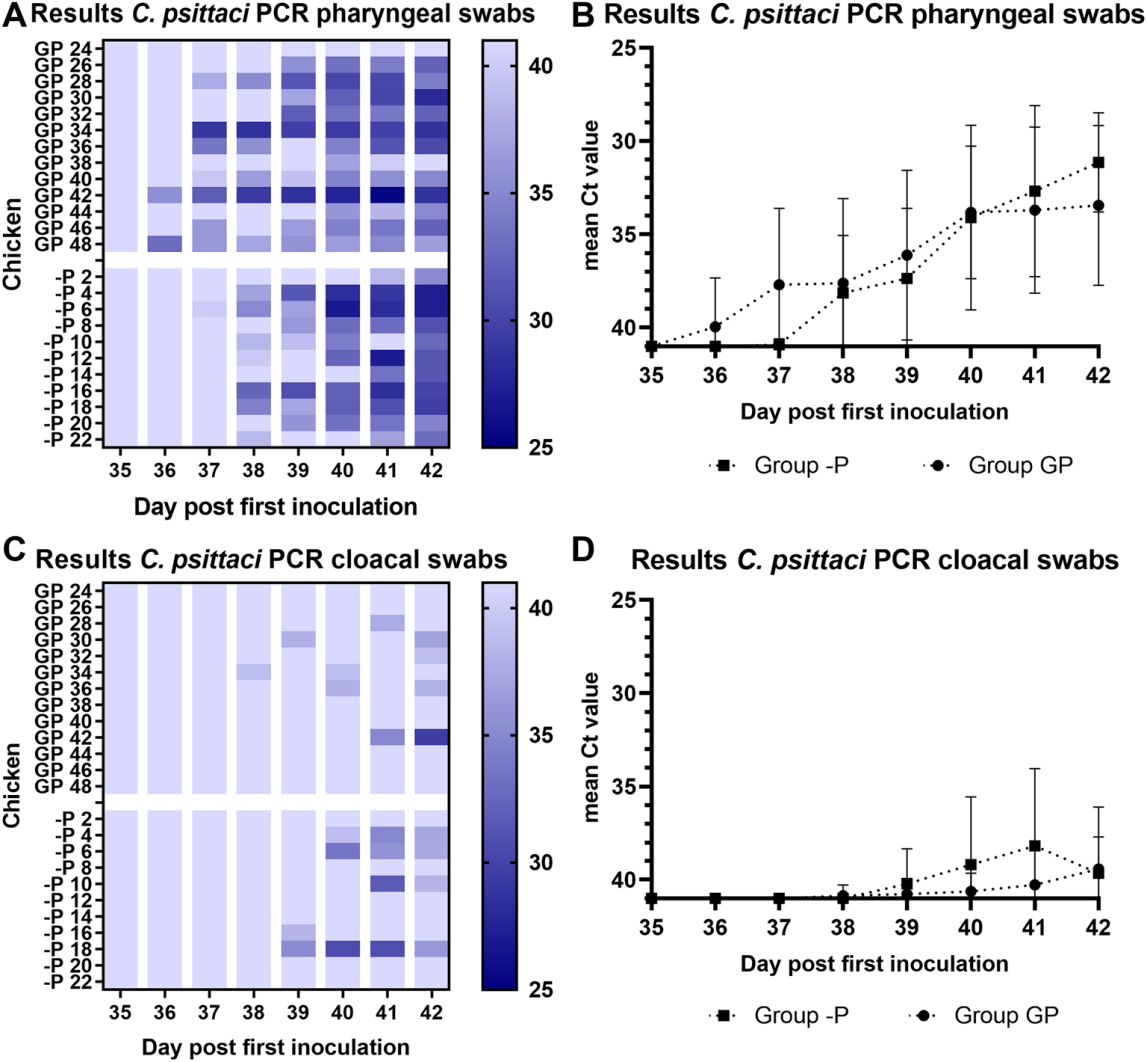
***C. psittaci qPCR results of group GP and -P***. In **A** individual results of the C. psittaci qPCR of pharyngeal swabs per timepoint are depicted in a heatmap. The darker the colour, the lower the Ct value as shown in the colour scale at the right side. Ct values > 40 or no qPCR signal are shown as Ct 41. **B** shows the mean Ct value of the pharyngeal swabs pet timepoint per group (of the *C. psittaci* qPCR). The error bar indicates the SD. On the Y-axis the cycle treshold (Ct) value is depicted. The Y-axis has been rotated and Ct values > 40 or no qPCR signal are shown as Ct 41. In **C** individual results of the *C. psittaci* qPCR of the cloacal swabs per timepoint are depicted in a heatmap. The darker the colour, the lower the Ct value as shown in the colour scale at the right side. Ct values > 40 or no qPCR signal are shown as Ct 41. **D** shows the mean Ct value of cloacal swabs in time per group (of the *C. psittaci* qPCR). The error bar indicates the SD. On the Y-axis the cycle treshold (Ct) value is depicted. The Y-axis has been rotated and Ct values > 40 or no qPCR signal are shown as Ct 41.

The *C. psittaci* inoculated groups (GP and -P) were tested with a *Chlamydiaceae* qPCR and a specific *C. psittaci* qPCR, which does not cross react with *C. gallinacea*. Before inoculation at day 35, 6/13 pharyngeal (46%, mean Ct 37.9) and 10/13 cloacal swabs (77%, mean Ct 34.6) of group GP test positive in the *Chlamydiaceae* qPCR, but negative in the *C. psittaci* qPCR (Figures 3A, C, Additional files 3A and B). This can be explained by the remaining presence of *C. gallinacea* NL_G47 and is in line with the findings at day 28 (Figures 2A, B). From day 36 onwards, the mean Ct value in the *Chlamydiaceae* qPCR in pharyngeal and cloacal swabs of group GP is lower than the mean Ct value in the *C. psittaci* qPCR (Additional file 3A and B). This difference seems to be caused by the remaining presence of *C. gallinacea* NL_G47 until day 37 in pharyngeal swabs and until day 38 in cloacal swabs, also when the results of group -P are taken into account. At the remaining days, a difference in sensitivity between both qPCRs might also play a role (Additional file 3). All individual qPCR results of pharyngeal and cloacal swabs are reported in Additional file 4.

After the second inoculation, qPCR based shedding of *C. psittaci* was higher in pharyngeal swabs as compared to cloacal swabs and based on the pharyngeal swabs, no significant difference between the groups was observed (Figure 3). At day 42, 11/13 (85%) pharyngeal swabs tested *C. psittaci* qPCR positive in group GP as compared to 11/11 (100%) in group -P (Figure 3A). In cloacal swabs, 4 chickens (31% in GP, 36% in -P) tested *C. psittaci* qPCR positive at day 42 in both groups (Figure 3C).

**Figure 4:**
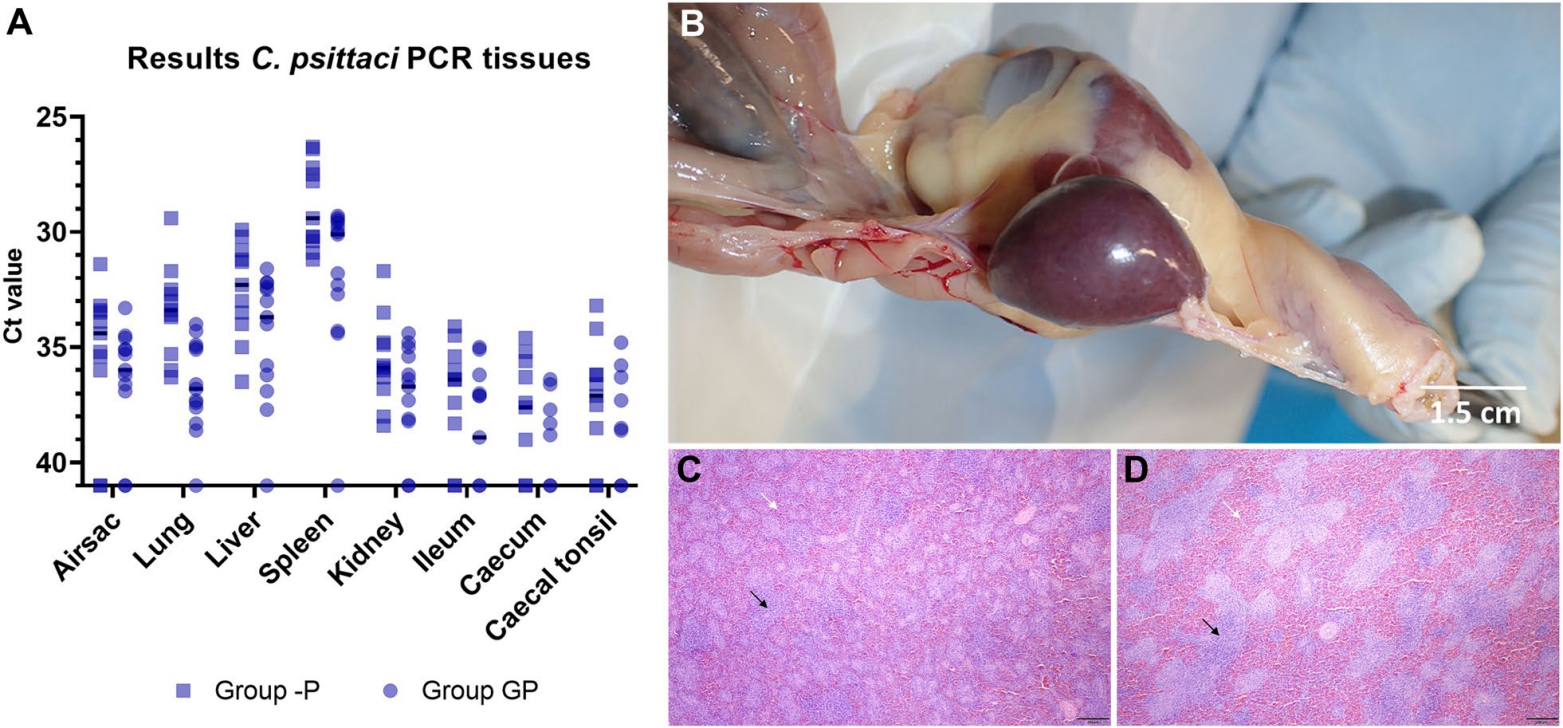
**Results of tissues in group GP and -P**. **A**: the *C. psittaci* qPCR results of tissue samples are shown. On the Y-axis the cycle treshold (Ct) value is depicted. The Y-axis has been rotated and Ct values > 40 or no qPCR signal are shown as Ct 41. The bar indicates the median. **B**: macroscopic enlargement of the spleen in a *C. psittaci* infected animal. **C** and **D**: histological examination of the spleen (same magnification, bar = 200 μm). Note the pronounced hyperplasia of the red (white arrows) and white pulp (black arrows) in the *C. psittaci* infected animal (**D**) compared to the animal infected with *C. gallinacea* (**C**).

In both groups, GP and -P, no clinical signs were observed during part 2 of the experiment based on the scoring card criteria. At necropsy, enlarged spleens were observed in 12/13 (92%) chickens of group GP and 10/11 (91%) of group -P. (Figure 4B) Histological examination of the spleen showed a marked hyperplasia of both white and red pulp. (Figure 4C) The hyperplasia of the white pulp included both the peri-arteriolar lymphocyte sheath (PALS) as well as the peri-ellipsoid sheath (PELS). In group -P one chicken showed diffuse small white spots on the liver. Histological examination revealed a multifocal hepatitis with small foci of coagulation necrosis and influx of heterophils. In another chicken the airsacs had a glazy appearance which was diagnosed by histopathology as an exudative aerosacculitis. The presence of chlamydial antigen however could not be confirmed with immunohistochemistry in any of the tissues examined.

In group GP, *C. psittaci* was detected by qPCR in 10/13 airsac samples (77%, median Ct 36), 12/13 lung samples (92%, median Ct 36.8), 12/13 liver samples (92%, median Ct 33.7), 12/13 spleen samples (92%, median Ct 30.1), 10/13 kidney samples (77%, median Ct 36.7), 7/13 ileum samples (54%, median Ct 38.9), 5/13 caecum samples (38%, median Ct 41) and 6/13 samples of the caecal tonsil (46%, median Ct 41). In group -P, *C. psittaci* was detected by qPCR in 9/11 airsac samples (82%, median Ct 34.4), 11/11 lung samples (100%, median Ct 33.4), 11/11 liver samples (100%, median Ct 32.3), 11/11 spleen samples (100%, median Ct 29.4), 11/11 kidney samples (100%, median Ct 35.9), 8/11 ileum samples (73%, median Ct 36.4), 7/11 caecum samples (64%, median Ct 37.6) and 8/11 samples of the caecal tonsil (73%, median Ct 37.1). Figure 4A shows the tissue dissemination patterns in group GP and -P overlapped. Overall there was a significant difference between the Ct values of the groups GP and -P (χ^2^ = 5.83, *p* = 0.016).

In group GP, one chicken remained *C. psittaci* qPCR negative in pharyngeal swabs, cloacal swabs, and tissue samples during the entire experiment.

#### Secondary inoculations with *C. gallinacea* NL_F725 (group GG and -G)

**Figure 5:**
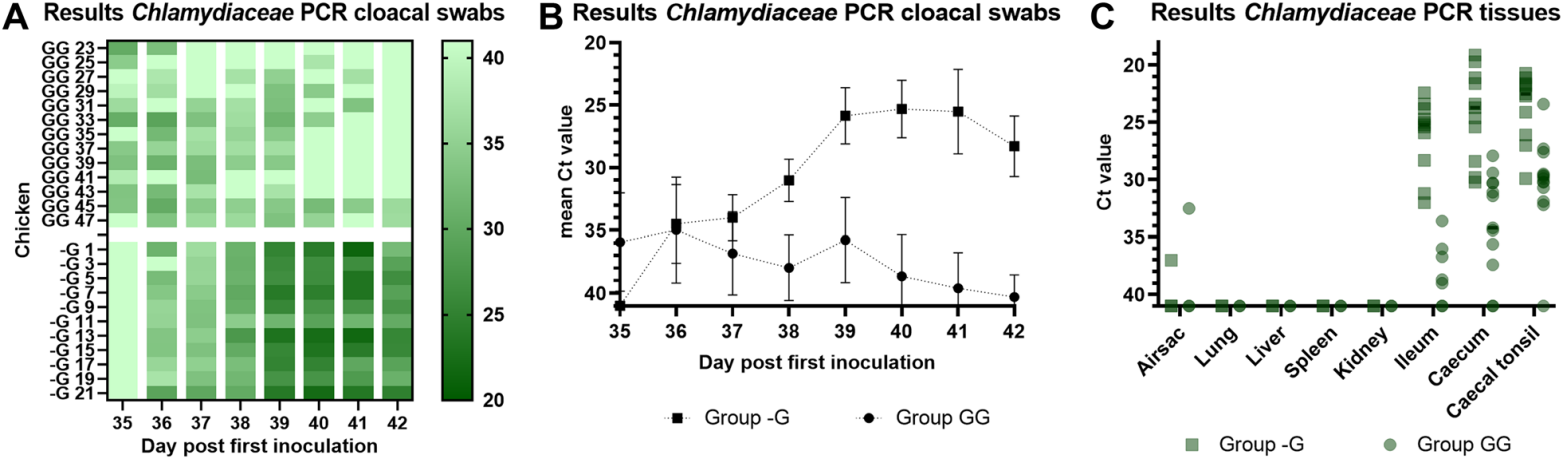
***Chlamydiaceae qPCR results of group GG and -G.*** In **A** individual results of the *Chlamydiacea* qPCR of the cloacal swabs per timepoint are depicted in a heatmap. The darker the colour, the lower the Ct value as shown in the colour scale at the right side. Ct values > 40 or no qPCR signal are shown as Ct 41. **B** shows the mean Ct value of cloacal swabs in time per group (of the *Chlamydiaceae* qPCR). The error bar indicates the SD. On the Y-axis the cycle treshold (Ct) value is depicted. The Y-axis has been rotated and Ct values > 40 or no qPCR signal are shown as Ct 41. In **C** the *Chlamydiacea* qPCR results of tissue samples are shown. The Y-axis has been rotated and Ct values > 40 or no qPCR signal are shown as Ct 41. The bar indicates the median.

Samples of the *C. gallinacea* NL_F725 infected groups (GG and -G) were only tested with the *Chlamydiacea* qPCR, because no strain specific real-time qPCR was available for *C. gallinacea* NL_F725 or NL_G47. In the group that was initially inoculated with *C. gallinacea* NL_G47 and subsequently inoculated with *C. gallinacea* NL_F725 (GG) significant reduced cloacal shedding was observed (Figures 5A, B) as compared to group -G (χ^2^ = 35.6, *p* < 0.001). In group GG qPCR based cloacal shedding decreased in time, but in the control group (-G) shedding increased (Figure 5B). At the end of the experiment at day 42, 2/13 (15%) cloacal swabs tested positive (Ct < 40) in group GG, while all (11/11) cloacal swabs in group -G tested positive (Figure 5A).

At necropsy no pathological lesions were observed in group GG and -G. In group GG 5/13 (38%) chickens tested qPCR positive in the ileum (median Ct 41), 10/13 (77%) in the caecum (median Ct 34.2) and 12/13 (92%) in the caecal tonsil (median Ct 29.9). In group -G, 11/11 (100%) chickens tested positive in the ileum, caecum and caecal tonsil (median Ct 25.2, 23.7 and 22.5 respectively) (Figure 4C). In both groups, GG and -G, one chicken tested positive in the airsac (Ct 32.5 and 37 respectively). All other tissue samples tested qPCR negative.

#### Differences between *C. psittaci *and *C. gallinacea* inoculation

**Figure 6:**
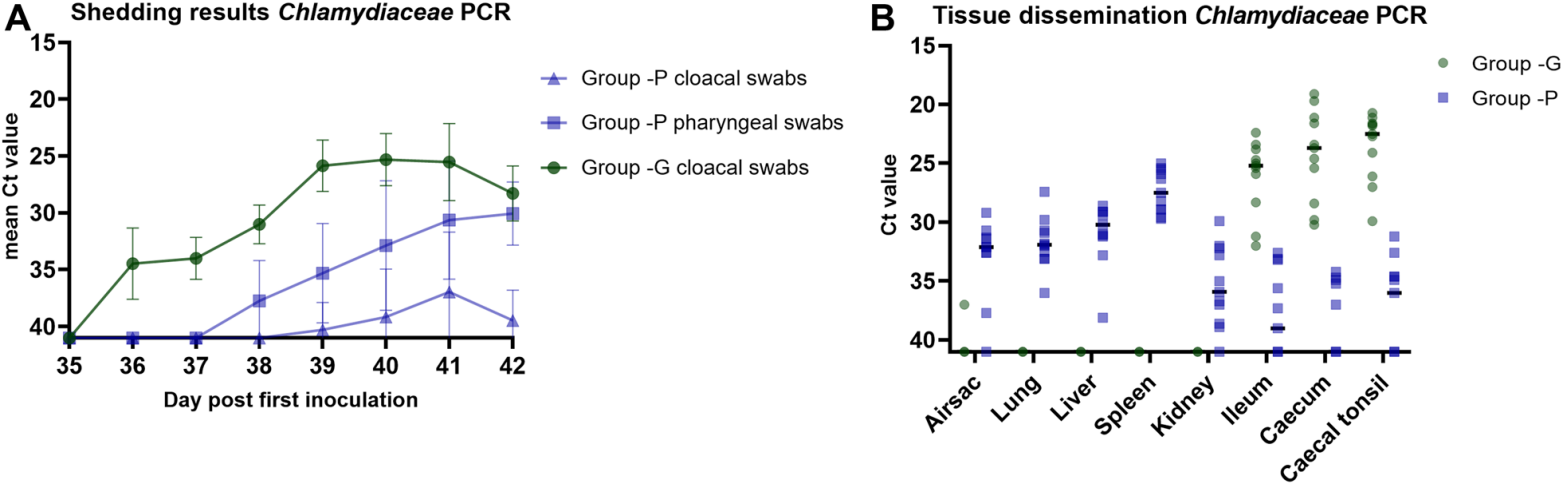
***Chlamydiaceae qPCR results of group -G and -P. A*** shows the mean Ct value of swabs in time per group of the *Chlamydiaceae* qPCR. The error bar indicates the SD. On the Y-axis the cycle treshold (Ct) value is depicted. The Y-axis has been rotated and Ct values > 40 or no qPCR signal are shown as Ct 41. In **B** the *Chlamydiaceae* qPCR results of tissue samples are shown of group -G and group -P. The Y-axis has been rotated and Ct values > 40 or no qPCR signal are shown as Ct 41. The bar indicates the median.

In the *C. gallinacea* inoculated control group (-G) and the *C. psittaci* inoculated control group (-P) the shedding and tissue dissemination pattern was different. In group -G cloacal shedding was higher than in group -P and the start of the excretion was different: the *C. gallinacea* inoculated group started shedding on day 1 post inoculation (day 36), while the *C. psittaci* inoculated group started shedding only on day 4 after inoculation (day 39) (Figure 6A). In group -P, shedding mainly occurred in pharyngeal swabs. In group -G pharyngeal swabs were not collected. In tissues *Chlamydia* was mainly detected in the ileum, caecum and caecal tonsil in all chickens of the -G group. In the -P group *Chlamydia* could be detected in all tissues, but the lowest Ct values were detected in the spleen and the highest in the gut in contrast to the results of the *-*G group (Figure 6B).

Summarising, chickens were succesfully infected with *C. gallinacea* NL_G47 in part 1 of the experiment while the controls remained *Chlamydia* negative. In part 2 no difference in *C. psittaci* shedding was observed between the group that was initially inoculated with *C. gallinacea* NL_G47 and subsequently infected with *C. psittaci* (GP) as compared to the control group that was subsequently inoculated with *C. psittaci* (-P). In the group that was initially inoculated with *C. gallinacea* NL_G47 and subsequently inoculated with *C. gallinacea* NL_F725 (GG) significant reduced cloacal shedding was observed as compared to the control group (-G). Furthermore, *C. psittaci* was mainly excreted via the pharyngeal and detected in systemic organs such as the spleen, while *C. gallinacea* was mainly detected in the gut. In none of the four groups clinical signs were observed based on the scoring card criteria.

## Discussion

In 2018, a high prevalence of *C. gallinacea* was detected on Dutch layer farms, but *C. psittaci* was absent in contrast to earlier studies in surrounding countries [6, 11, 13]. We hypothesised that the absence of *C. psittaci* could possibly be explained by cross protection between *C. gallinacea* and *C. psittaci*. This idea was also driven by the fact that cross reactive antibodies between chlamydial species are known to occur, because of the close structural similarity among some of the major surface antigens [15]. To investigate whether an infection with *C. gallinacea* could protect against an infection with *C. psittaci*, chickens were first inoculated with *C. gallinacea* NL_G47 and subsequently inoculated with either *C. gallinacea* NL_F725 or with *C. psittaci* NL_Borg. The inoculations did not result in a difference in shedding or tissue dissemination of *C. psittaci* between the group that did not receive a first inoculation and the group that did receive a first inoculation with *C. gallinacea*, so cross protection was not observed.

We did observe a difference in tissue dissemination and shedding pattern between the groups that were inoculated with *C. gallinacea* and *C. psittaci*. In both groups the inoculation route was slightly different: the *C. gallinacea* groups were inoculated orally which resulted in an infection of the gut, while the *C. psittaci* groups received an oro-nasal inoculation that caused a more systemic infection (i.a. of the spleen). In *C. gallinacea*, the oral route is the main route of transmission for *C. gallinacea* and transmission via the respiratory route could not be proven [9]. Considering *C. psittaci,* infections via the respiratory route are more efficient than infections via the oral route [25]. The different porte d’entree and subsequent localization of the infection is probably caused by a difference in tissue tropism of *C. gallinacea* and *C. psittaci*.

A difference in tissue tropism between *C. gallinacea* and *C. psittaci* could also partially explain why cross protection was not observed. The successful clearance of a *Chlamydia* infection depends on both a local and systemic, cell-mediated and humoral response with neutralizing antibodies that act either by inhibiting binding to epithelial cells or activation of complement, leading to lysis of the *Chlamydia* membrane [26, 27]. In our study, a rise in anti-*Chlamydia* serum antibodies was measured after the first inoculation with *C. gallinacea*, but, after the second inoculation, *C. psittaci* could be detected in organs such as the spleen and liver, suggesting circulating neutralizing antibodies against *C. psittaci* were not elicited or could not prevent infection. In a *C. trachomatis* vaccine study, neutralizing antibodies against the variable domain 4 (VD4) of MOMP were very important in preventing infection in a mouse model, but this effect was also specific [26]: small differences in the amino acid sequence of the epitope could already prevent neutralization [28, 29]. Additional in-vitro studies are therefore needed to investigate if *C. gallinacea* infection elicits neutralizing antibodies and if these antibodies have a neutralizing effect on *C. psittaci*.

If neutralizing antibodies are not elicited or do no neutralize *C. psittaci*, (partial) cross protection against *C. psittaci* would depend on local immune responses. However, a local response might not be effective because of the observed difference in tissue tropism between *C. gallinacea* and *C. psittaci*. In the group that received a first inoculation with *C. gallinacea* NL_G47 and a subsequent inoculation with *C. gallinacea* NL_F725 cross protection was observed. This could support the possible role of the local immune response, although neutralizing antibodies could have an effect as well. As already concluded, this would require in-vitro studies to investigate neutralizing antibodies and additional studies into the local immune response via the measuring of local IgA antibodies or transcriptomic analyses in the gut.

In addition, the difference in shedding pattern between *C. gallinacea* and *C. psittaci* could cause differences in transmission, which might alternatively explain why *C. gallinacea* was highly prevalent and *C. psittaci* was not detected in the prevalence study [6]. At first, the degree of shedding of *C. gallinacea* appeared to be higher in this study which will facilitate transmission and, second, the main route of transmission is different. In chickens, the respiratory route is likely to be more important for *C. psittaci* based on previous studies that compared inoculation routes and the higher degree of pharyngeal shedding in this study [25]. The degree of pharyngeal shedding might also depend on the stage of infection and the *C. psittaci* genotype or strain. In previous studies it was shown pharyngeal shedding was mainly higher during the early part infection until day eight [30]; our experiment ended at seven days post inoculation with *C. psittaci*. Furthermore, previous studies with *C. psittaci* genotype D resulted in more severe clinical disease and higher excretion than infections with genotype B [13, 30]. *C. psittaci* genotype A, C, D and E/B have been associated with chickens [31]. Therefore, comparative transmission studies with *C. gallinacea* and different genotypes of *C. psittaci* in chickens would be of added value to further understand how differences in infection dynamics could affect prevalence. These studies should also take into account sampling strategy regarding the differences in shedding pattern.

Before the second inoculation with *C. psittaci* or *C. gallinacea* NL_F725, 10/13 chickens where still shedding *Chlamydia* in both groups. It was expected shedding would decrease after transport to a clean environment, because it was thought part of the shedding might be explained by passive transfer or re-infection of *C. gallinacea* (DNA) from the environment. However, this effect was not observed and the transport of the chickens as a stress factor might have had an enhancing effect on cloacal shedding as known for *C. psittaci* [32].

The remaining presence of NL_G47 at the start of the second part of the experiment could have underestimated the effect on shedding in the GG group, because NL_G47 and NL_F725 could not be differentiated with the *Chlamydiaceae* qPCR. On the other hand, it could also have caused a type of competitive exclusion in which the local presence of NL_G47 prevented NL_F725 to enter gut epithelial cells [33]. This kind of effect seems unlikely, because it has not been described before in *Chlamydia* and might have been observed in both the GG and GP group. However, cloacal shedding and colonization of the gut in *C. psittaci* infection (group -P) was in general much lower than in *C. gallinacea* infection (-G) possibly due to a difference in tissue tropism as discussed above.

In group GP, one chicken remained *C. psittaci* qPCR negative in pharyngeal swabs, cloacal swabs, and tissue samples during the entire experiment. We do not have a clear explanation for this observation, but we hypothesize it might be caused by biological variation or heterogeneity in disease susceptibility or outcome between animals even though they have the same background. Heterogeneity in disease outcome and shedding of *C. psittaci* is observed in the field and known from other bacterial infections such as tuberculosis [34]. Furthermore, Figures 3A, C show there is variation in shedding between animals in both the GP and -P group. Therefore, it seems unlikely the chicken remained *C. psittaci* negative as a result of protection. There are also no indications the inoculation failed.

In conclusion, a prior *C. gallinacea* infection, does partially protect against a new *C. gallinacea* infection based on the qPCR based results of cloacal shedding. However, a prior infection with *C. gallinacea* is not protective against a subsequent infection with *C. psittaci* based on shedding and tissue dissemination. The absence of *C. psittaci* in an earlier prevalence study [6] can therefore not be explained by such cross protection. The question remains how often *C. psittaci* is introduced in chickens flocks, how well infections can be transmitted and whether infections might go unnoticed as no clinical signs were observed during our experiment. This would require future comparative transmission studies.

## Supplementary Information

**Additional file 1. Scoring card clinical signs.**

**Additional file 2. Differences in weight in group G and—(control).** Differences in weight (grams) in group G and group—(control) at the start of part 1 of the experiment and after 28 days is shown in a boxplot. The whiskers plot down to the smallest value and up to the largest and the box extends from the 25th to 75th percentile.

**Additional file 3. Differences between the Chlamdyiaceae qPCR and C. psittaci qPCR.** A and B show the mean Ct value of pharyngeal and cloacal swabs in time of group GP in the *Chlamydiaceae* qPCR and *C. psittaci* qPCR. C and D show the mean Ct value of pharyngeal and cloacal swabs in time of group -P in the *Chlamydiaceae* qPCR and *C. psittaci* qPCR. The error bar indicates the SEM in all figures. On the Y-axis the cycle treshold (Ct) value is depicted. The Y-axis has been rotated and Ct values > 40 or no qPCR signal are shown as Ct 41.

**Additional file 4. qPCR data of individual pharyngeal and cloacal swabs.**
