# Supplementary material for: Experimental Chlamydia gallinacea infection in chickens does not protect against a subsequent experimental Chlamydia psittaci infection
Source: Vet Res. 2021 Nov 20;52:141. doi: 10.1186/s13567-021-01011-y (PMC8605536; doi:10.1186/s13567-021-01011-y)
Supplement: Supplementary file 1 — Additional file 1. Scoring card clinical signs. [file 13567_2021_1011_MOESM1_ESM.docx]

# S1: Scoring card clinical signs

|  | No signs (0) | Mild (1) | Severe (2) |
| --- | --- | --- | --- |
| Mental state | Active, makes noise, responds to environment or handling | Less active, bulging (with feathers upright), but responding to environment | No response to environment, lying, retreating, hardly to no response to handling, stopped eating and drinking |
| Head | No discharge from nose or eye, no red eyes | Watery to mucous discharge from eye and/or nose (tear stripe), red eyes | Severe mucous or bloody discharge and/or dense red, swollen eyes |
| Upper airways | No sneezing or shaking with the head | Occasionally sneezing | Frequent sneezing and shaking of the head |
| Lower airways | No increased respiration frequency or symptoms of shortness of breath | Slightly increased respiration freq and / or noises such as gargling and grating | Clearly increased breathing frequency, open mouth, stretched neck, symptoms of shortness of breath, noises such as gargling and rattling |
| Gait and balance | Normal gait, no uncoordinated movements or tremors | Difficulty with coordination when standing up, can walk but seems to have more difficulty with coordination of movements | Disturbed balance, difficulty walking or paralysis, twisted neck, walking in circles, severe muscle tremors |
| Feces | Normal chicken feces, no abnormal consistency or color | Feces with abnormal color (green to yellow) and/or consistency (wetter) | Feces with abnormal color, consistency and quantity, presence of blood |
