# Supplementary figures and images for: Experimental Chlamydia gallinacea infection in chickens does not protect against a subsequent experimental Chlamydia psittaci infection
Source: Vet Res. 2021 Nov 20;52:141. doi: 10.1186/s13567-021-01011-y (PMC8605536; doi:10.1186/s13567-021-01011-y)

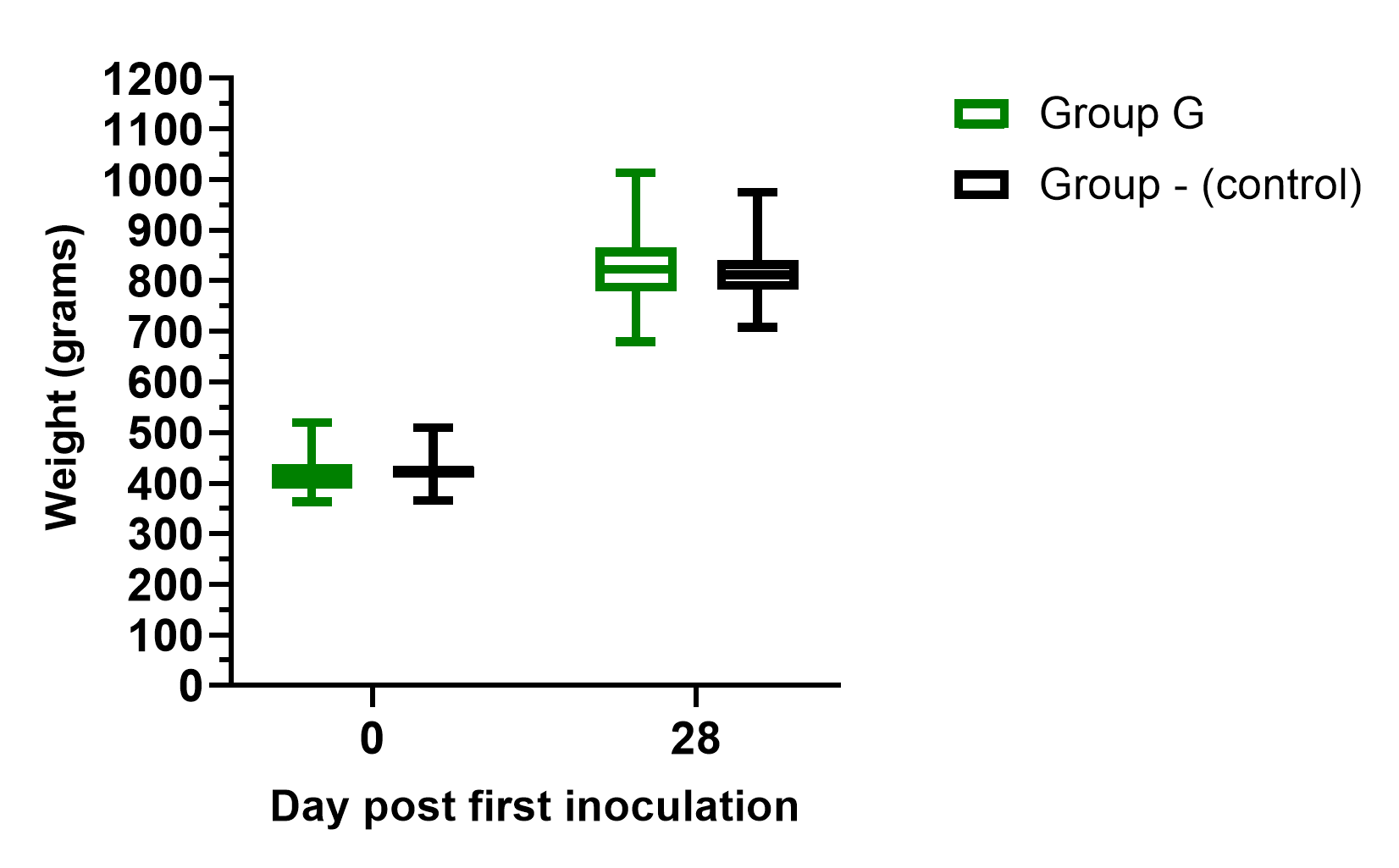

Supplement: Supplementary file 2 — Additional file 2. Differences in weight in group G and—(control). Differences in weight (grams) in group G and group—(control) at the start of part 1 of the experiment and after 28 days is shown in a boxplot. The whiskers plot down to the smallest value and up to the largest and the box extends from the 25th to 75th percentile. [file 13567_2021_1011_MOESM2_ESM.tif]

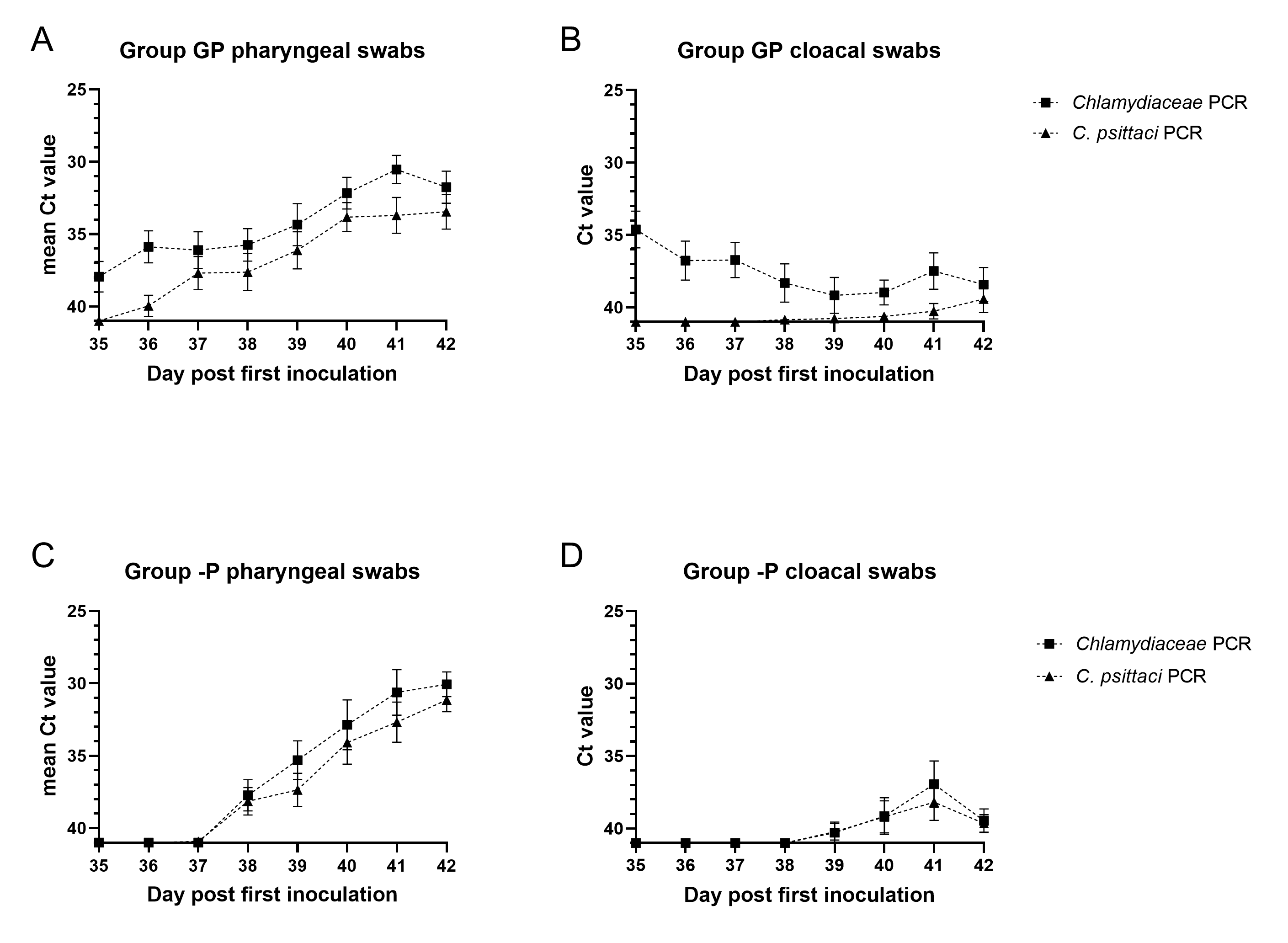

Supplement: Supplementary file 3 — Additional file 3. Differences between the Chlamdyiaceae qPCR and C. psittaci qPCR. A and B show the mean Ct value of pharyngeal and cloacal swabs in time of group GP in the Chlamydiaceae qPCR and C. psittaci qPCR. C and D show the mean Ct value of pharyngeal and cloacal swabs in time of group -P in the Chlamydiaceae qPCR and C. psittaci qPCR. The error bar indicates the SEM in all figures. On the Y-axis the cycle treshold (Ct) value is depicted. The Y-axis has been rotated and Ct values > 40 or no qPCR signal are shown as Ct 41. [file 13567_2021_1011_MOESM3_ESM.tif]
